# The social context in bark beetle – fungus bioassays: a case study in European fir engraver bark beetles and their fungal associates

**DOI:** 10.3389/fmicb.2025.1717396

**Published:** 2026-01-16

**Authors:** Sifat Munim Tanin, Jon Andreja Nuotclà, Peter H. W. Biedermann

**Affiliations:** Chair of Forest Entomology and Forest Protection, Albert-Ludwigs-Universität Freiburg, Stegen, Germany

**Keywords:** fungal volatiles, *Geosmithia* sp., *Ophiostoma piceae*, *Pityokteines curvidens*, *Pityokteines vorontzowi*

## Abstract

**Background:**

Certain species of bark beetles (Curculionidae: Scolytinae) are among the most aggressive herbivorous forest insects due to their mass aggregation behavior and symbiosis with filamentous fungi. These characteristics help them overwhelm the natural defenses of the healthy trees they attack, and consequently, they are classified as primary pest species. Despite their important role in the beetles’ success, the community of fungal symbionts and their key mutualist taxa are only well understood for a few symbionts in a small number of bark beetle species. Recent developments have shown that key mutualists can be identified using *in vitro* olfactory or gustatory bioassays. However, these assays have only tested mixed-sex groups of beetles. This introduces potential biases compared to individual assays due to the known tendency of these beetles to aggregate.

**Methods:**

This study focuses on the poorly studied fungal symbionts of European fir bark beetles in the genus *Pityokteines*, specifically *P. vorontzowi* and *P. curvidens*. We used a newly developed, two-tier bioassay to evaluate the attraction of beetles to olfactory and gustatory fungal cues in a specific order to identify essential mutualists. Additionally, we are the first to investigate whether testing individual beetles or mixed- or same-sex groups influences the outcome of such bioassays.

**Results:**

Our results show that *Pityokteines* beetles responded more strongly to physical contact with the fungus than to volatiles alone. Of the five commonly isolated species, only *Geosmithia* sp. and *Ophiostoma piceae* were attractive. Females responded to volatile cues, while males did not. Both sexes preferred to bore their feeding tunnels in these two fungi but were repelled by one of the other species, *Graphilbum fragrans*. The social context significantly impacted the beetles’ behavior: same-sex groups exhibited the strongest response to the offered fungal cues, while mixed-sex groups demonstrated the weakest response.

**Conclusion:**

In summary, we identified key fungal species in *Pityokteines* bark beetles that now need to be assessed individually for their function(s). Most importantly, our results suggest that previous studies should be reassessed because sex and social context must be considered when conducting such bioassays.

## 1 Introduction

Bark beetles (Scolytinae) are a specious group of weevils that feed and build their breeding tunnels (i.e., galleries) in the phloem-cambium layer of typically unhealthy or already dead trees (Kirkendall et al., 2015). Some species, such as facultative tree-killing, conifer-attacking species, are currently on the rise in temperate forests and are causing major forest damage as climate change reduces tree health at landscape scales (Hlásny et al., 2021; Netherer et al., 2024). Decades of research have shown that bark beetles are able to proliferate and overwhelm tree defenses with the help of symbiotic fungi that can, for example, detoxify tree chemistry, provide additional nutrition and block water flow (Hofstetter et al., 2015; Krokene, 2015). These beneficial symbionts are typically ascomycete fungi of the unrelated orders Ophiostomatales, Microascales and Hypocreales (Kirisits, 2004; Harrington, 2005; Biedermann and Vega, 2020). In addition to these mutualists, bark beetle galleries typically host other commensal or antagonistic fungal symbionts (Six, 2012). Despite their important role in beetle fitness, the community of fungal symbionts, the key taxa and in particular their role is only known for a few symbionts in a small number of bark beetle species, such as the economically important *Ips* and *Dendroctonus* species (Kirisits, 2004; Six, 2012). Knowing the key fungal symbionts and their roles for each species is not only important for understanding the biology of bark beetles, but also for developing novel monitoring and management tools (Gugliuzzo et al., 2021).

Determining the role of fungal symbionts for bark beetles is not straightforward. A first step is usually to test which fungi are vertically transmitted by the dispersing beetles and inoculated into newly colonized trees (Six, 2003). Many bark beetle species have mycetangia, evolved cuticular spore-bearing organs that selectively transfer spores of beneficial fungal symbionts from the natal nest during dispersal (Francke-Grosmann, 1956; Mayers et al., 2022). Transmitted communities can be described after identifying the core microbial associations with bark beetles using culture-dependent, culture-independent, or preferably both methods simultaneously (Baños-Quintana et al., 2024). The role of beneficial symbionts has typically been inferred from fungal phylogeny (i.e., belonging to a known group of beneficial taxa) and/or their presence in beetle mycetangia (Hulcr et al., 2020). Testing beetle attraction or repellence has proven to be the easiest and most fruitful approach to quickly classify the role of fungal symbionts (Hulcr et al., 2011; Diehl et al., 2023; Kandasamy et al., 2023; Nuotclà and Taborsky, 2024). The responses of adult beetles and even larvae toward individual fungal or bacterial species have been assessed by recording their responses to olfactory and gustatory cues in bioassay arenas (Tanin et al., 2021; Diehl et al., 2023).

However, many bark beetle-fungus bioassays have been conducted under rather artificial, not well controlled setups. For instance, individual beetles have been tested for either their olfactory or their gustatory response, but never in combination, even though in nature beetles have access to both cues in close succession. Importantly, most studies have used mixed-sex groups in bioassays with fungi (Kandasamy et al., 2019; Tanin et al., 2021), despite bark beetles’ known tendency to aggregate. They are textbook examples on how to coordinate mass attacks using aggregation pheromones (Blomquist et al., 2010). Therefore, effects of fungal volatiles may be interfered with or overshadowed by conspecific pheromones introducing follower effects (Nuotclà and Taborsky, 2024). Both imprecisions may have greatly confounded the results and so it is unclear if some of the conclusions still hold if sexed beetles are individually tested.

European fir bark beetles (FBBs) in the genus *Pityokteines* (*P. curvidens, P. spinidens, P. vorontzowi*) are currently on the rise in several parts of southern and central Europe due to climate change decreasing the health of silver fir (*Abies alba*; Durand-Gillmann et al., 2014; Parisi et al., 2025). However, given their low economic importance in the past, all three species are poorly studied and fungal symbionts have been only described by culturing methods in a few populations (Jankowiak and Kolařík, 2010). The key fungal symbionts are only hypothesized, because so far studies on their role and attraction to the beetles are missing.

In this case study on FBBs we first aimed to develop a bioassay that allows testing not only olfactory, but also gustatory responses of adult bark beetles toward previously isolated core fungal associates. Through this approach, we next aimed to address the following key questions: (1) Are *P. vorontzowi* and *P. curvidens* attracted to associated fungi through fungal volatile cues or direct gustatory contact? (2) Do male and female *P. vorontzowi* exhibit differences in their attraction to associated fungi? (3) Does the social context affect male or female responses; i.e., if they are tested individually, in same-sex or mixed-sex groups?

## 2 Materials and methods

### 2.1 Collection of beetles

Naturally infested silver fir branches and logs were collected from the Black Forest (47°53’00”N 7°45’35”E) in Freiburg im Breisgau, Germany, at an elevation of 400–500 m on June 18th, 2022. Branches and logs were cut into 50–70 cm pieces. 4–7 branches or one log were hung in each emergence trap, with a funnel underneath connected to a collection vial for beetle collection. Twenty-two emergence traps were prepared to collect enough beetles for regular bioassay experiments. Beetles started emerging immediately after hanging the traps in the experimental forest of the institute (Chair of Forest Entomology and Protection, University of Freiburg, Germany). Emerging beetles were sorted by sex. Before releasing beetles into the bioassay arena, every beetle was checked carefully to ensure their ability to walk independently. If the beetles were not used immediately, they were stored in vials on moist, sterile filter paper in the fridge (5 °C) for a maximum of 5 days.

### 2.2 Preparation of bark agar media

Freshly cut silver fir logs were collected from the institute’s forest. The outer bark was removed, and the inner phloem was peeled using a sharp knife for bark media preparation. The inner bark was then cut into small pieces (2–5 mm) using scissors. A total of 96 g of sliced bark (12% of the media) was soaked in 300 ml of distilled water in a glass vial overnight. The following day, the bark-water mixture was ground using the Springlane Hanno high-performance mixer (2000 watt), creating a bark-water paste. This paste was transferred into a 1000 ml autoclave vial, along with 24 g of agar (3% of the media) and 380 ml of additional distilled water. The vial was mixed thoroughly and autoclaved (at 121 °C for 20 min) for the experiment.

### 2.3 Fungi cultivation in bark agar media

Seven different fungi were cultivated on the bark agar media to assess the behavior of *P. vorontzowi* (Table 1). Five of these fungi were tested in a two-tier behavioral bioassay. Four were evaluated in two-choice arenas in a single petri dish containing mixed-sex pairs of male and female beetles, and seven were tested in choice arenas containing individual beetles. In parallel, two fungi were cultivated to assess the behavior of *P. curvidens* using a two-tier behavioral bioassay. The fungi were selected based on their common association with FBBs, as determined by the number of colony-forming units (CFUs) on Yeast Extract Malt Agar (YEMA) media, as well as on the basis of published works by others (Jankowiak et al., 2017; Jankowiak and Bilański, 2018).

**TABLE 1:** Fungi were evaluated in three different bioassay setups for *P. vorontzowi* and *P. curvidens*.

Fungi	Beetles	Two-tier behavioral bioassay	Two-choice single petri dish arena with male-female pair	Two-choice single petri dish arena with single beetle
*Geosmithia* sp.	*P. vorontzowi*	80 (63)	100 (88)	100 (87)
	*P. curvidens*	80 (59)	–	–
*Ophiostoma piceae*	*P. vorontzowi*	80 (67)	100 (88)	100 (92)
	*P. curvidens*	80 (67)	–	–
*Cladosporium* sp.	*P. vorontzowi*	80 (66)	100 (89)	100 (77)
*Graphilbum fragrans*	*P. vorontzowi*	80 (60)	100 (77)	100 (94)
*Talaromyces rugulosus*	*P. vorontzowi*	–	–	100 (69)
*Penicillium bialowiezense*	*P. vorontzowi*	80 (52)	–	100 (70)
*Beauveria bassiana*	*P. vorontzowi*	–	–	100 (94)
	Total	560 (434)	400 (342)	700 (583)

The table summarizes the total number of beetles tested in each setup, with males and females equally represented. Numbers in parentheses indicate the final number of beetles after removing irresponsive or dead individuals (=final sample size).

Depending on the specific experimental design, fungi were cultivated in 90 mm or 60 mm Petri dishes. For growing fungi in Petri dishes, immediately after pouring and allowing the bark agar media to solidify in the Petri dishes, we textured each dish with crisscrossing straight lines using a sterile metal tweezer, facilitating beetle movement across the media. For each experiment, we used actively growing 6–7 days old fungal cultures. In 90 mm Petri dishes, two fungal plugs were inoculated, while in 60 mm Petri dishes, a single fungal plug was inoculated.

### 2.4 Behavioral bioassays

#### 2.4.1 Two-tier behavioral bioassay

##### 2.4.1.1 Preparation of two-tier behavioral bioassay

**FIGURE 1:**
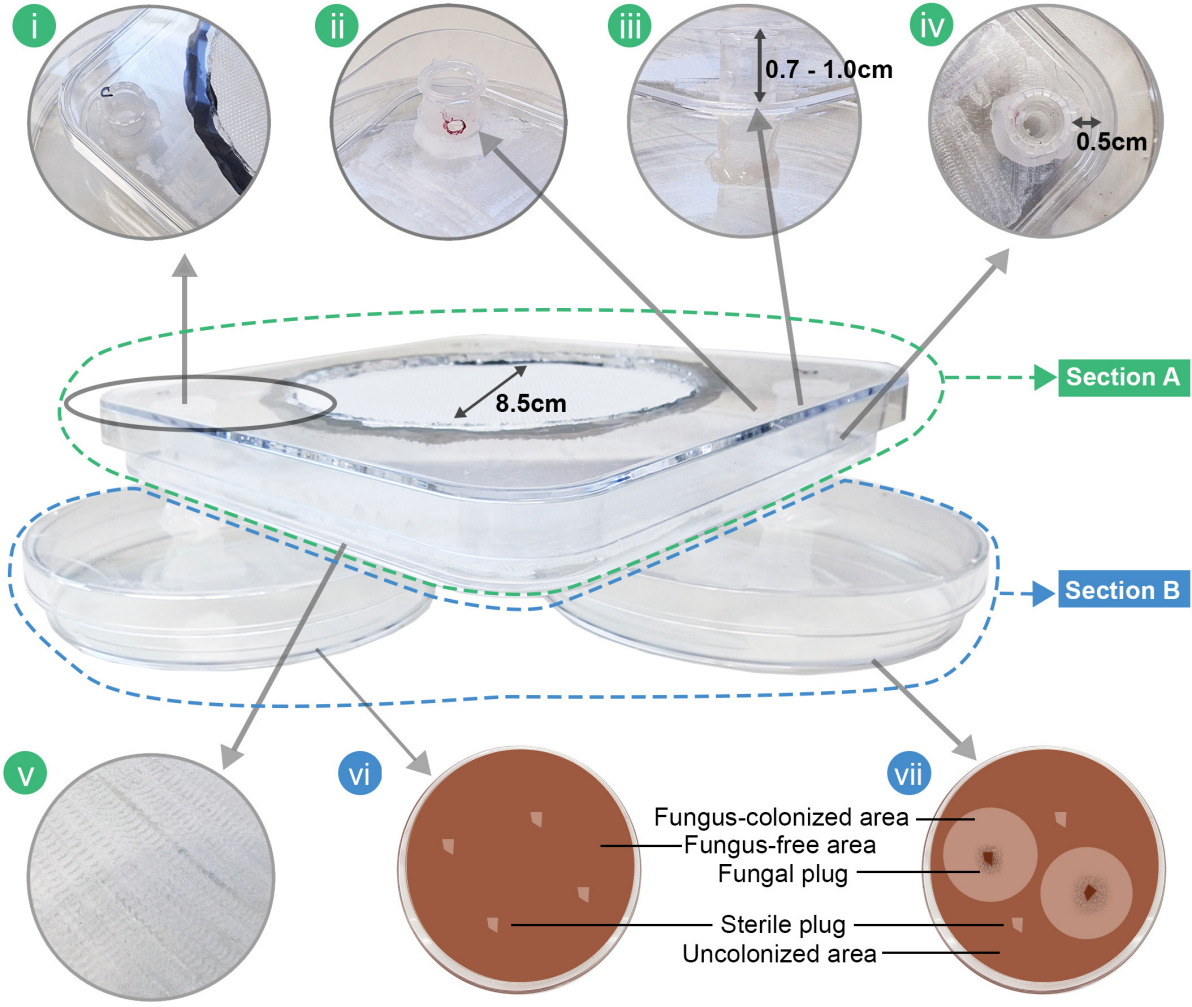
Two-tier behavioral bioassay used to evaluate beetle responses to fungi. The arena consists of two main sections: Section A (outlined in green) features a central square chamber with a textured floor to facilitate beetle movement **(v)**. The chamber includes two entrances constructed from modified Eppendorf tubes **(i)**, each fitted with four holes to allow beetle entry **(ii)**. The height of the Eppendorf tubes from the chamber floor is approximately 0.7–1.0 cm **(iii)**, and they are positioned 0.5 cm inward from each corner of the chamber **(iv)**. Section B (outlined in blue) comprises two 90 mm Petri dishes placed beneath the chamber, containing either fungus-free **(vi)** or fungus-colonized bark agar media **(vii)**.

After testing multiple two-choice behavioral bioassays in a preliminary study, a new design, termed a two-tier behavioral bioassay, was constructed to align with beetle behavior, optimizing experimental results based on the size and activity of the targeted beetles. This bioassay enabled to monitor two distinct beetle behaviors simultaneously: (1) response to volatile cues (bioassay section A) and (2) response to gustatory cues (bioassay section B; Figure 1).

In section A (Figure 1), the bioassay arena was constructed using 120 × 120 × 17 mm Petri dishes (Greiner bio-one, Cat no: 688102). The inner bottom surface was roughened with a Dremel 2001 (Dremel Deutschland) to help the beetles walk easily in the arena (Figure 1v). Two holes were made 0.5 cm (Figure 1iv) from the edge of the Petri dish to fit 1.5 ml Eppendorf tubes, which were tightly inserted and glued from the outside to prevent volatile interference. The lids of the Eppendorf tubes were removed to allow airflow and volatiles to enter the arena. The height of the Eppendorf tubes was kept 0.7–1.0 cm (Figure 1iii) above the arena surface of the Petri dish. A large circular hole (∅ 8.5 cm) was cut in the Petri dish lid, leaving enclosed spaces (Figure 1i) in the corners where the tubes were attached. This modification was made to develop a higher saturated source of fungal volatile near the Eppendorf tubes, helping the beetles locate the volatile source. A circular nylon mesh was glued to close the hole in the Petri dish lid to prevent beetles from escaping and to maintain airflow in the arena. Four additional holes (Figure 1ii) (∅ 3–4 mm) were made in the Eppendorf tubes roughly 1 mm above the inner bottom surface as entrances for the beetles to crawl into the Eppendorf tube. The bottom of the Eppendorf tubes was cut to create a 5–6 mm opening.

Section B contains two 90 mm Petri dishes as sources of volatile cues: one with fungal growth on bark agar media (Figure 1vii) and the other with fungus-free bark agar media (Figure 1vi). The lids of the dishes were modified by cutting holes in the center and attaching them to the bottoms of Eppendorf tubes, so each lid could be securely closed onto a dish to connect section A. When beetles enter the tubes in response to volatile cues, they fall onto one of the two Petri dishes (Section B) where they can make a second choice based on direct access to the media or fungus (i.e., gustatory cues), respectively. Beetles that reach the fungal-colonized dish encounter two zones in the medium: one with fungus and one without. In contrast, beetles that reach the fungus-free dish only have the option to dig into the fungus-free media. Textured surfaces of the media enable beetles to move easily and select their preferred digging site. Their digging locations were then recorded to evaluate their response to gustatory cues.

##### 2.4.1.2 Execution of two-tier behavioral bioassay

A total of 140 individual bioassay experiments were conducted in seven batches (Table 1). For each batch, we prepared 20 bioassays, each offering a choice between active fungus-growing bark agar media and fungus-free bark agar media. All bioassays were placed under a laboratory fume cupboard (*maxXima Niedrigraum TA*, wrt–Laborbau GmbH & Co., KG; airflow 660 m^3^ h^–1^; 1998) for continuous air circulation. Following a pilot experiment testing activity levels with either four or eight beetles per arena, we chose to release four beetles in each arena. Half of the bioassays were tested with only male beetles and the remaining half with only female beetles. In odd-numbered bioassays, the fungal volatile source was positioned on the right side, while in even-numbered bioassays, it was placed on the left side. This alternating arrangement helped minimize any directional bias in beetle movement. We placed four sterile plugs in the fungus-free bark agar media, and two additional sterile plugs in the fungal bark agar media to mimic the presence of plugs in both treatments. After releasing the beetles into the arenas, we closed the laminar flow hood and covered it with black plastic to eliminate light pollution, allowing the experiment to run overnight (11–12 h) in complete darkness. The following day, beetle behavior was recorded in two stages. First, we recorded the number of beetles that reached either the fungus-colonized Petri dish or the fungus-free Petri dish as olfactory response. In the second stage, we examined how many beetles dug into the media as gustatory response.

#### 2.4.2 Choice arena with *P. vorontzowi* (male-female pair or single beetle): response to gustatory cues

**FIGURE 2:**
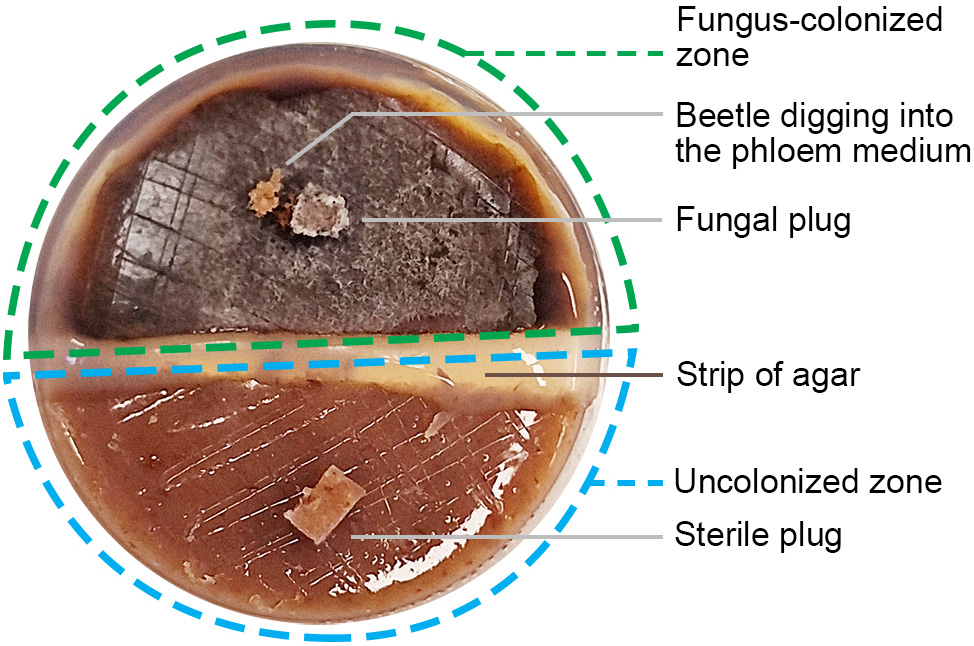
A two-choice bioassay in a single Petri dish offers a fungus-colonized zone and an uncolonized zone for beetles. A sterile plug was introduced to the uncolonized zone to mimic the fungal plug in the fungus-colonized zone.

To further evaluate beetle responses to specific fungal associates and increasing the sample size for gustatory responses in a efficient manner, we established two-choice arenas using 60 mm Petri dishes (Figure 2). In each arena, half of the medium was colonized by an actively growing fungal culture, while the other half contained uncolonized media, following the method of Kandasamy et al. (2019). To mimic the fungal plug present in the colonized half, a sterile plug was placed in the center of the uncolonized zone. A 3–4 mm straight strip of 3% pure agar was used to connect the two zones.

For paired trials, four fungal species were tested: *Geosmithia* sp., *O. piceae*, *Cladosporium* sp., and *Graphilbum fragrans*. One male and one female beetle were released into each arena, which was then sealed with parafilm to prevent escape. Fifty replicates were prepared for each fungus and incubated overnight (10–11 h) in a dark climate chamber at 25 °C. The following day, beetles were removed from the tunneling holes for sex determination, and the number of holes in the fungus-colonized and uncolonized zones were recorded.

For single-beetle trials, the same arena design was used, testing the above four fungi along with three additional species: *Penicillium bialowiezense*, *Talaromyces rugulosus*, and *Beauveria bassiana*. Each fungus was tested in 100 arenas: 50 with male beetles and 50 with female beetles.

### 2.5 Statistics

We analyzed responses of beetles in groups of four same sexed beetles in two stages: olfactory response (fungus vs. control) and subsequent digging behavior as gustatory response (dug in vs. found on the surface). Beetles that did not decide in the olfaction stage were excluded from all analyses and individuals arriving alone on a side were coded as “single individual,” while two or more beetles on the same side were coded as “same-sex group” when arriving in the gustatory response stage. The data of the gustatory response were combined with the results from the single petri dish set up.

Olfactory choice data from the two-tier assay were analyzed with generalized linear mixed-effects models (GLMMs) with a binomial error distribution and logit link. Fixed effects included beetle species and fungus species, and a random intercept for observation accounted for the non-independence of beetles tested within the same arena.

Digging behavior was analyzed as a binary response using GLMMs. For interspecific comparisons, we fitted a model restricted to two fungi (*O. piceae* and *Geosmithia* sp.) tested in both beetle species (*P. vorontzowi* and *P. curvidens*), with beetle species as fixed effect and observation as random intercept. To test whether digging propensity depended on sex and the social context (tested as single individual, mixed sex pair or same sex group) we restricted analyses to *P. vorontzowi* and compared the digging propensity of beetles on control phloem medium to the digging propensity in fungus-colonized media. Estimated marginal means (EMMs) with 95% confidence intervals were obtained from fitted models, and for fungus choice in the olfaction stage, pairwise contrasts were evaluated on the log-odds scale with Tukey adjustment for multiple comparisons. To determine statistical deviance from random choice, we compared EMMs of choice probabilities to the null expectation of 0.5 by computing standardized z-scores (*z* = (estimate – 0.5)/SE) and corresponding two-tailed *p*-values, assuming asymptotic normality of the estimates. All analyses were conducted in RStudio (Posit team, 2024) with R version 4.4.2 (R Core Team, 2024), using the dplyr (Wickham et al., 2023), emmeans (Lenth, 2025), ggplot2 (Wickham, 2016), lme4 (Bates et al., 2015), tidyr (Wickham et al., 2024), and writexl (Ooms, 2025) packages.

## 3 Results

### 3.1 Response of FBBs to fungal volatiles

**FIGURE 3:**
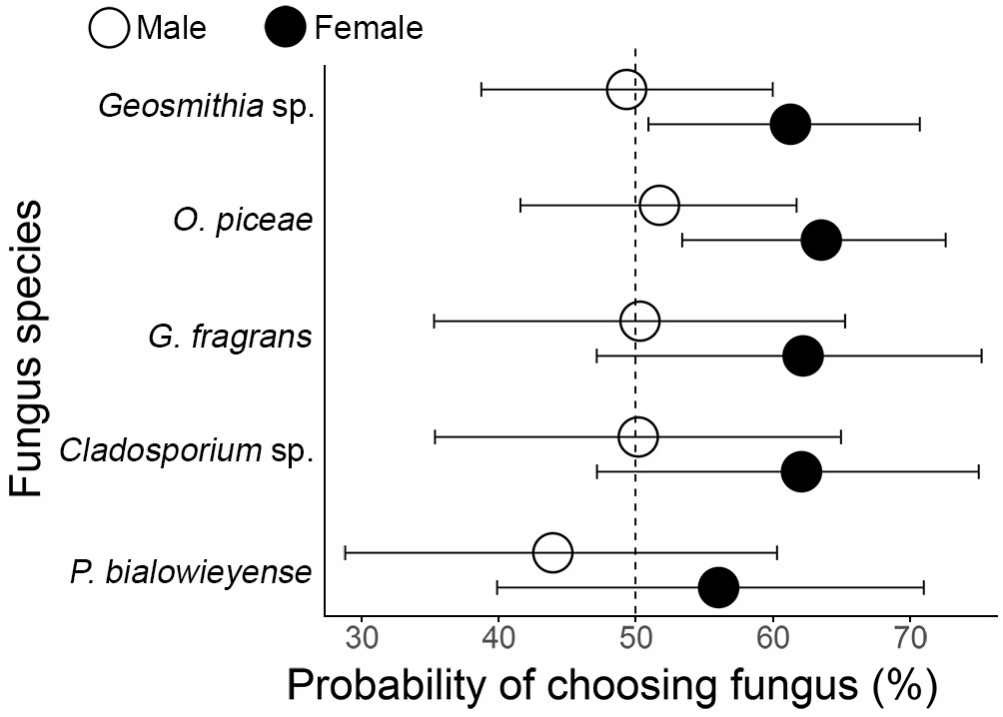
Probability of beetles choosing different fungal species during the olfaction stage of the two-tier behavioral bioassay. Estimated marginal means (EMMs) with 95% confidence intervals were obtained from a generalized linear mixed-effects model (GLMM) with fungus species, beetle species, and sex as fixed effects and observation as a random intercept. Male and female beetles are represented by different fill colors (black = female, white = male). The dashed vertical line at 50% indicates equal probability of choosing fungus versus choosing control. Confidence intervals (horizontal whiskers) reflect uncertainty around the estimated probabilities. Significant differences were observed only for females, which were significantly choosing *O. piceae* (*p* = 0.006) and *Geosmithia* sp. (*p* = 0.027) more often than random choice and had a slightly higher overall propensity to choose fungi compared to males (*p* = 0.018).

Beetles generally showed no strong preference for specific fungal volatiles. None of the fungal species significantly differed from each other in terms of olfactory attraction (corrected for multiple comparison, all *p* > 0.1). Likewise, there was no difference in olfactory choice between *P. curvidens* and *P. vorontzowi* (GLMM: Estimate = –0.005, *p* = 0.98). However, sex significantly influenced choice: males were less likely than females to choose the fungal side (Estimate = –0.49, *p* = 0.018; Figure 3). Interestingly, females showed a significantly above-random choice for both *Geosmithia* sp. (*p* = 0.027) and *O. piceae* (*p* = 0.006), whereas their olfactory responses to the other fungi and those of males to all fungi did not differ from random expectation (*p* > 0.05; Figure 3).

### 3.2 Response of FBBs to physical contact with the fungi

**FIGURE 4:**
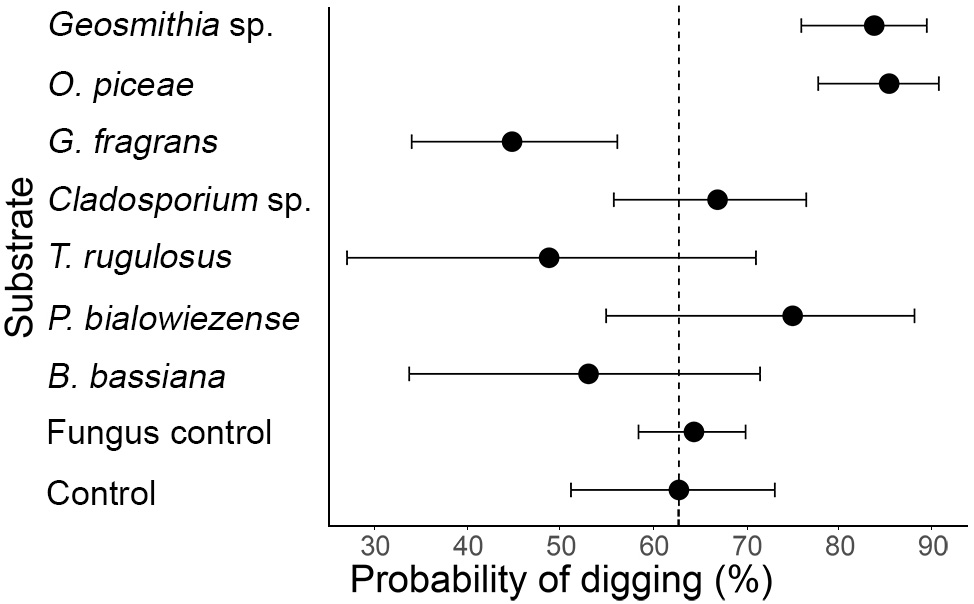
Probability of digging by *P. vorontzowi* on different substrates in single- and group-context arenas. Points show estimated marginal means (EMMs) from a generalized linear mixed-effects model (GLMM) with chosen side, sex, social context, and their interaction as fixed effects, and observation as a random intercept. Horizontal whiskers represent 95% confidence intervals. The dashed vertical reference line indicates the digging probability on the fungus-free section of the two-tiered olfactometer setup (Control), which was used as the reference in our models. This allows direct visual comparison of digging propensity on different fungi and control media. Beetles dug significantly more often into *O. piceae* (*p* < 0.001) and *Geosmithia* sp. (*p* = 0.002). than reference control, whereas *G. fragrans* elicited significantly less digging (*p* = 0.035). The propensity of beetles to dig into the fungus-free zone of the gustatory setup is labeled here as “fungus control” and did not differ statistically from the reference (*p* > 0.1).

**FIGURE 5:**
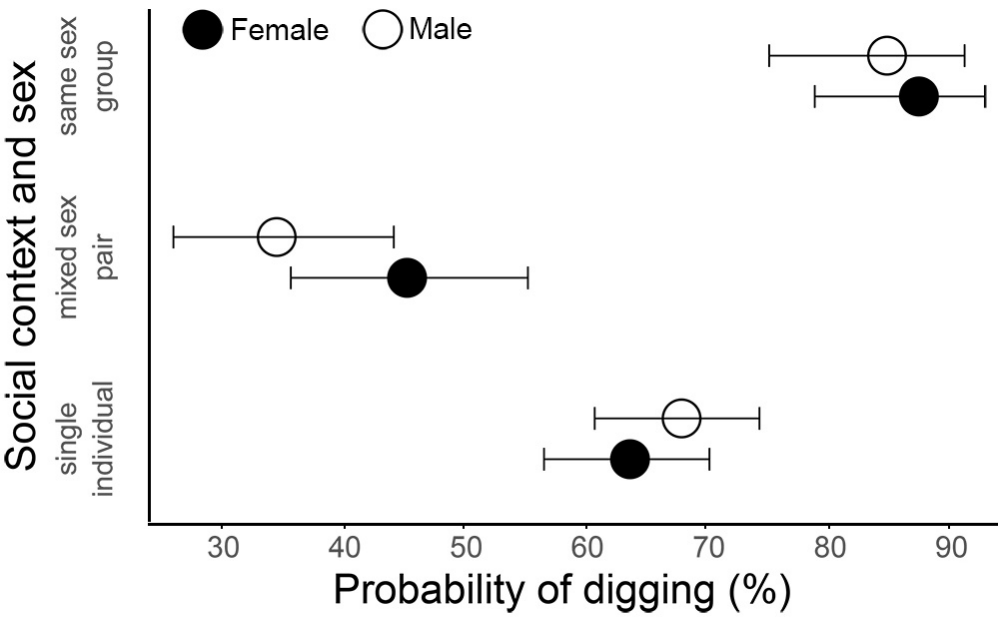
Effect of social context and sex on the probability of digging by *Pityokteines vorontzowi*. Estimated marginal means (EMMs) with 95% confidence intervals were derived from a generalized linear mixed-effects model (GLMM) with chosen substrate, sex, social context, and their interaction as fixed effects, and observation as a random intercept. Social context is shown as single individuals, mixed-sex pairs, and same-sex groups. Males and females are represented by different fill colors (black = female, white = male). Digging probability increased in same-sex groups for both sexes (*p* < 0.001) and decreased in mixed-sex pairs (*p* = 0.001; particularly in males *p* interaction sex and social context = 0.037), relative to single beetles. Confidence intervals (horizontal whiskers) indicate uncertainty around the estimated probabilities.

Digging propensity in *P. vorontzowi* depended on fungus species, social context, and to a lesser extent sex (Figures 4, 5 and Table 2). Compared to the fungus-free section, tunneling was significantly more likely for beetles on *Geosmithia* sp. (GLMM: Estimate = 1.116, *p* = 0.002) and *O. piceae* (Estimate = 1.238, *p* < 0.001). By contrast, *G. fragrans* elicited less digging than in the control (Estimate = –0.730, *p* = 0.035). No significant effects were detected for *Cladosporium* sp., *T. rugulosus, P. bialowiezense* or *B. bassiana* (all *p* > 0.1). Beetles found on the control side (the fungus-free section) of the two-tier set-up were as likely to have dug as those found on the control side of the single Petri dish set-up (Estimate = 0.070, *p* > 0.1). When compared to each other in the digging stage of the two-storied essay, *P. curvidens* and *P. vorontzowi* differed significantly in their propensity to dig. *P. curvidens* had a higher probability of digging compared to *P. vorontzowi* (Estimate = –0.74 for *P. vorontzowi*, *p* = 0.004).

**TABLE 2:** Fixed-effect estimates from a generalized linear mixed-effects model (GLMM) examining the probability of digging by the beetle species *P. vorontzowi.*

Fixed effects	Estimate	SE	*Z*-value	*P*-value
(Intercept)	−0.67	0.331	−2.026	0.043
**Medium type (control side in olfactory setup vs.__)**				
Control in gustatory setup	0.070	0.284	0.246	0.806
*Beauveria bassiana*	−0.400	0.479	−0.835	0.404
*Penicillium bialowiezense*	0.575	0.516	1.114	0.265
*Talaromyces rugulosus*	−0.570	0.545	−1.045	0.296
*Cladosporium* sp.	0.182	0.350	0.520	0.603
*Graphilbum fragrans*	**−0.730**	**0.347**	**−2.106**	**0.035**
*Ophiostoma piceae*	**1.238**	**0.365**	**3.393**	**<0.001**
*Geosmithia* sp.	**1.116**	**0.360**	**3.099**	**0.002**
Sex (male)	0.189	0.187	1.007	0.314
**Social context (alone vs. __)**				
Mixed sex pair	**−0.749**	**0.236**	**−3.176**	**0.001**
Same sex group	**1.374**	**0.334**	**4.118**	**<0.001**
**Interaction sex × Social context (alone female vs. male compared to __)**				
Mix sexed female vs. male	**−0.639**	**0.307**	**−2.081**	**0.037**
Same sexed female vs. male	−0.407	0.439	−0.928	0.354

The model included the type of substrate (phloem control of the olfaction part in the two-tier behavioral bioassay as reference) sex (female as reference), social context (single individual as reference), and the interaction between sex and social context as fixed effects, with the test arena included as a random intercept to account for non-independence within beetles that where tested together. Estimates are presented on the logit scale, with standard errors, *z*-values, and significance levels indicated. Significant effects are highlighted in bold font (*p* < 0.05).

### 3.3 Effects of social context on beetle choice

Social context strongly modulated digging behavior in *P. vorontzowi*. Same-sex groups were substantially more likely to dig than single individuals (GLMM: Estimate = 1.37, *p* < 0.001; Figure 5 and Table 2), while beetles tested in mixed-sex pairs were less likely to dig than singles (Estimate = –0.75, *p* = 0.001). A significant interaction indicated that males in mixed-sex pairs were particularly unlikely to dig (Estimate = –0.64, *p* = 0.037). Sex alone had no significant main effect on digging (Estimate = 0.19, *p* = 0.31).

## 4 Discussion

To optimize the assessment of behavioral responses and identification of candidate fungal mutualists, we designed a new, two-tiered bioassay. This bioassay integrates the attraction of beetles to olfactory and gustatory cues within a single experimental run, while enabling the evaluation of each cue separately. Previous studies have used olfactory (Kandasamy et al., 2019; Tanin et al., 2021; Diehl et al., 2023; Nuotclà and Taborsky, 2024) or gustatory bioassays (Kandasamy et al., 2019; Tanin et al., 2021) to test beetle preference for certain fungi. In our setup, we can collect beetles within a narrow emergence window to address multiple research questions simultaneously. This setup overcomes the common challenge of securing sufficient replicates during the short flight period and reduces the risk of behavioral variation under different climatic conditions. When assessing gustatory responses through digging behavior, our setup offers two control scenarios: one in which the beetles may be influenced by the physical presence of the fungi and one in which they are not. Additionally, this bioassay is cost-effective and simple to construct in large quantities, enabling the testing of large groups of beetles simultaneously. In our study, we combined the results of the two-tier bioassay with those of the two-choice bioassay in a single Petri dish since these can be replicated with less effort. This allowed us to test sufficient numbers of beetles in a gustatory scenario to analyze beetle behavior jointly and, for the first time, test whether bark beetle preferences are influenced by social context and sex.

In the two-tier bioassay and the two-choice assays in single Petri dishes when beetles are in physical contact with the fungus and control media, we focused exclusively on digging behavior. We considered this the most reliable indicator of beetle choice in contrast to individuals that were simply roaming within the arena. We emphasized digging behavior as a primary response to gustatory cues, albeit it may be mediated by a combination of both olfactory and gustatory responses. Additionally, several other studies have successfully used digging as a proxy for preference for, and the beneficial function of, a certain fungal symbiont for the beetles (Kandasamy et al., 2019; Tanin et al., 2021). In pilot experiments, we observed that nearly half of the beetles did not dig into the medium during an overnight trial. However, the percentage of digging increased with longer durations. To maintain consistency, we restricted our assays to overnight experiments and instead increased the sample size to achieve reliable results.

Unlike earlier studies which reported strong responses in bark beetles to fungal volatiles (Kandasamy et al., 2019; Tanin et al., 2021; Jirošová et al., 2022), only females of the species *P. vorontzowi* and *P. curvidens* weakly responded to two associated fungi (*Geosmithia* sp. and *O. piceae*), while the remaining fungi (*G. fragrans*, *Cladosporium* sp. and *P. bialowiezense*) were neither attractive nor avoided. This suggests that these bark beetle species rely minimally on fungal volatiles to locate suitable hosts. Given the evident sex-specific differences, it is more likely that females respond to fungal cues to locate males. In polygamous bark beetles such as *Pityokteines* spp., males are typically the first colonizers (Borden, 1974). They initiate breeding by tunneling, introducing fungi into their galleries and producing aggregation pheromones (Harring, 1978). Females may use aggregation pheromones and fungal volatiles to locate freshly excavated entrance holes and males. In the European spruce bark beetle (*Ips typographus*), it has been shown that fungal symbionts produce some of the substances that attract males and females and lead to aggregation (Kandasamy et al., 2016; Zhao et al., 2019). Our results indicate that in *Pityokteines* spp. females are more sensitive to fungal volatiles than males, which corroborates the idea that females are attracted to the fungus present in galleries that males initiated. It is likely that some defensive plant chemistry (e.g., terpenoids) is degraded by the fungi to produce the compounds present in their volatile bouquet (Zhao et al., 2019; Kandasamy et al., 2023).

However, physical contact with the fungi is crucial. Notably, *O. piceae* and *Geosmithia* sp. promoted digging when in physical contact, while *G. fragrans* reduced it, indicating that *O. piceae* and *Geosmithia* sp. are key mutualists. This is consistent with earlier studies on *Pityokteines* spp. and *Cryphalus piceae*, which documented associations with *Geosmithia* spp., *O. piceae* and *G. fragrans*. Several *Geosmithia* species and *O. piceae* were reported as dominant (Jankowiak and Kolařík, 2010; Jankowiak et al., 2017; Jankowiak and Bilański, 2018). Conversely, the presence of the well-known entomopathogenic fungus *B. bassiana* did not elicit a response that differed from the control, which may explain its frequent association with many bark beetle species. As a specialist pathogen it may be able to hide from the beetles (Diehl et al., 2023). The pathogenicity of *B. bassiana* against bark beetles remains controversial, with studies reporting either high mortality (Kreutz et al., 2004; Olatinwo et al., 2018) or minimal effects under natural conditions, likely due to the influence of various biotic and abiotic factors (Mann and Davis, 2021). Overall, these contrasting responses reinforce the view that some fungi act as mutualists, enhancing colonization, whereas others may function as commensals or even antagonists/competitors.

Compared to *P. vorontzowi*, *P. curvidens* exhibited a stronger response to fungi when in physical contact with *Geosmithia* sp. and *O. piceae*. Both beetle species typically co-occur on dying and dead silver fir trees and both are commonly associated with these fungal species (Jankowiak and Kolařík, 2010; Jankowiak et al., 2017; Jankowiak and Bilański, 2018), possibly suggesting horizontal transfer. However, while *P. vorontzowi* is found more in the crown and branches, *P. curvidens* is slightly larger and colonizes the stem and thicker branches (Nierhaus-Wunderwald, 1995). This difference in host material preference may be related to the importance of fungal symbionts, as *Ips typographus*, which colonizes stems of Norway spruce (*Picea abies*), also shows a stronger dependence on its fungal symbionts than *Pityogenes chalcographus*, which is mostly found in the crowns (Schebeck et al., 2023).

Despite clear evidence of sex-specific roles in bark beetle ecology and known tendency to aggregate (Harring, 1978), most behavioral assays so far, used mixed-sex groups when testing responses to fungal attraction but did neither report whether sex ratio nor beetle numbers influenced the outcomes (Kandasamy et al., 2019; Tanin et al., 2021). To our knowledge we are the first to test single males and females, same sex and mixed sex groups and our results highlight that both sex and social group can substantially influence outcomes. Digging activity was significantly higher in arenas containing groups of two to four same-sex beetles compared to single individuals or mixed male-female pairs. This pattern may reflect competitive interactions among beetles of the same sex. Although no studies have directly demonstrated that bark beetles accelerate tunneling as beetle density increases, previous work has shown that some species are aware of conspecifics’ initial tunnels and maintain certain distances to ensure successful colonization (Byers, 1989; Grosman et al., 1992). Within our bioassay arenas, spatial restriction likely limited the beetles’ ability to maintain normal spacing, while confinement may have imposed stress that accelerated the onset of tunneling. Consequently, the proximity of conspecifics could have enhanced digging activity, either through competitive interactions or mutual stimulation. In contrast, single individuals of either sex dug more frequently than mixed male-female pairs, suggesting that courtship interactions between sexes may divert beetles from digging. Unfortunately, we do not have behavioral observations how individuals interacted with each other on the plates.

Overall, this study contributes to the expanding body of research emphasizing the complexity of bark beetle-fungus interactions, highlighting the necessity of considering multiple behavioral pathways when evaluating these interactions (Six and Klepzig, 2021). Our approach, which integrates olfactory and gustatory responses and explicitly accounts for sex and group size, provides a more nuanced understanding of how European fir engraver bark beetles interact with their fungal partners. Our findings emphasize that not all fungal associates play equivalent roles: while some facilitate colonization, others appear neutral or even inhibitory. Future studies should integrate chemical ecology and GC–MS with detailed analyses of sex and group composition, investigating potential pheromone production by males and females in bioassay conditions (Harring, 1978). If beetles produce pheromones in bioassays, bioassays involving combinations of males and females or *P. vorontzowi* and *P. curvidens* could reveal interesting patterns of attraction or competition. Such efforts will refine our knowledge of beetle–fungus ecology and inform strategies for monitoring and managing the impact of bark beetles on silver fir forests.

## 5 Conclusion

Our study demonstrates that *P. vorontzowi* and *P. curvidens* exhibited relatively weak responses to fungal volatiles. However, gustatory responses, as measured by digging behavior, revealed a strong response toward *O. piceae* and *Geosmithia* sp., while other fungi either had no effect or repelled the beetles. These contrasting patterns suggest that fungal associates play diverse ecological roles, ranging from mutualists to commensals and competitors. By introducing a novel two-tier bioassay and accounting for sex and group size, we provide a more robust framework for evaluating beetle-fungus interactions. Together, our findings emphasize the importance of considering both olfactory and gustatory pathways, as well as experimental design factors, in order to fully understand the functional roles of fungal symbionts in bark beetle ecology.

## Data Availability

The original data presented in the study are included in the Supplementary Tables 1, 2. Further inquiries can be directed to the corresponding authors.
