# Supplementary material for: The social context in bark beetle – fungus bioassays: a case study in European fir engraver bark beetles and their fungal associates
Source: Front Microbiol. 2026 Jan 16;16:1717396. doi: 10.3389/fmicb.2025.1717396 (PMC12855431; doi:10.3389/fmicb.2025.1717396)
Supplement: Supplementary file 2 [file Table_2.docx]

arena: identifier for the bioassay in which beetles were tested at the same time.

social: mixed=pair of female and male, alone=single individual male or female tested, group=four same sexed individuals tested at once

sex: male or female beetle

choice: in case of two-tier setup (experiment=olfaction) 1=fungus side, 0=control side, NA=did not choose (dead, not found, or still roaming arena); in case of single plate setup (experiment=gustatory) 1=fungus side, 0=control side, NA=dead or not found

dig: 1=dug in, 0=roaming on surface

fungus.spec: fungus species

beetle.spec: beetle species

experiment: gustatory=single petri dish setup, olfactoric=two-tier setup

fungusArea: inside=in two-tier experiment, beetles that choose the fungus side and are found inside the area with fungus, outside=in two-tier experiment, beetles that choose the fungus side and are found outside the area with fungus (so in the "fungus control")
